# Expression Atlas update: insights from sequencing data at both bulk and single cell level

**DOI:** 10.1093/nar/gkad1021

**Published:** 2023-11-22

**Authors:** Nancy George, Silvie Fexova, Alfonso Munoz Fuentes, Pedro Madrigal, Yalan Bi, Haider Iqbal, Upendra Kumbham, Nadja Francesca Nolte, Lingyun Zhao, Anil S Thanki, Iris D Yu, Jose C Marugan Calles, Karoly Erdos, Liora Vilmovsky, Sandeep R Kurri, Anna Vathrakokoili-Pournara, David Osumi-Sutherland, Ananth Prakash, Shengbo Wang, Marcela K Tello-Ruiz, Sunita Kumari, Doreen Ware, Damien Goutte-Gattat, Yanhui Hu, Nick Brown, Norbert Perrimon, Juan Antonio Vizcaíno, Tony Burdett, Sarah Teichmann, Alvis Brazma, Irene Papatheodorou

**Affiliations:** European Molecular Biology Laboratory, European Bioinformatics Institute, EMBL-EBI, Hinxton CB10 1SD, UK; European Molecular Biology Laboratory, European Bioinformatics Institute, EMBL-EBI, Hinxton CB10 1SD, UK; European Molecular Biology Laboratory, European Bioinformatics Institute, EMBL-EBI, Hinxton CB10 1SD, UK; European Molecular Biology Laboratory, European Bioinformatics Institute, EMBL-EBI, Hinxton CB10 1SD, UK; European Molecular Biology Laboratory, European Bioinformatics Institute, EMBL-EBI, Hinxton CB10 1SD, UK; European Molecular Biology Laboratory, European Bioinformatics Institute, EMBL-EBI, Hinxton CB10 1SD, UK; European Molecular Biology Laboratory, European Bioinformatics Institute, EMBL-EBI, Hinxton CB10 1SD, UK; European Molecular Biology Laboratory, European Bioinformatics Institute, EMBL-EBI, Hinxton CB10 1SD, UK; European Molecular Biology Laboratory, European Bioinformatics Institute, EMBL-EBI, Hinxton CB10 1SD, UK; European Molecular Biology Laboratory, European Bioinformatics Institute, EMBL-EBI, Hinxton CB10 1SD, UK; European Molecular Biology Laboratory, European Bioinformatics Institute, EMBL-EBI, Hinxton CB10 1SD, UK; European Molecular Biology Laboratory, European Bioinformatics Institute, EMBL-EBI, Hinxton CB10 1SD, UK; European Molecular Biology Laboratory, European Bioinformatics Institute, EMBL-EBI, Hinxton CB10 1SD, UK; European Molecular Biology Laboratory, European Bioinformatics Institute, EMBL-EBI, Hinxton CB10 1SD, UK; European Molecular Biology Laboratory, European Bioinformatics Institute, EMBL-EBI, Hinxton CB10 1SD, UK; European Molecular Biology Laboratory, European Bioinformatics Institute, EMBL-EBI, Hinxton CB10 1SD, UK; European Molecular Biology Laboratory, European Bioinformatics Institute, EMBL-EBI, Hinxton CB10 1SD, UK; European Molecular Biology Laboratory, European Bioinformatics Institute, EMBL-EBI, Hinxton CB10 1SD, UK; European Molecular Biology Laboratory, European Bioinformatics Institute, EMBL-EBI, Hinxton CB10 1SD, UK; Cold Spring Harbour Laboratory, One Bungtown Road, Cold Spring Harbor, NY 11724, USA; Cold Spring Harbour Laboratory, One Bungtown Road, Cold Spring Harbor, NY 11724, USA; Cold Spring Harbour Laboratory, One Bungtown Road, Cold Spring Harbor, NY 11724, USA; USDA ARS NEA, Plant Soil & Nutrition Laboratory Research Unit, Ithaca, NY 14853, USA; FlyBase-Cambridge, Department of Physiology, Development and Neuroscience, University of Cambridge Downing Street, Cambridge CB2 3DY, UK; Perrimon Lab, Department of Genetics, Harvard Medical School, Boston MA 02115, USA; FlyBase-Cambridge, Department of Physiology, Development and Neuroscience, University of Cambridge Downing Street, Cambridge CB2 3DY, UK; Perrimon Lab, Department of Genetics, Harvard Medical School, Boston MA 02115, USA; FlyBase-Harvard Biological Laboratories, Harvard University, 16 Divinity Avenue, Cambridge, MA 02138, USA; European Molecular Biology Laboratory, European Bioinformatics Institute, EMBL-EBI, Hinxton CB10 1SD, UK; European Molecular Biology Laboratory, European Bioinformatics Institute, EMBL-EBI, Hinxton CB10 1SD, UK; Wellcome Trust Sanger Institute. Wellcome Genome Campus, Hinxton CB10 1SA, UK; European Molecular Biology Laboratory, European Bioinformatics Institute, EMBL-EBI, Hinxton CB10 1SD, UK; European Molecular Biology Laboratory, European Bioinformatics Institute, EMBL-EBI, Hinxton CB10 1SD, UK

## Abstract

Expression Atlas (www.ebi.ac.uk/gxa) and its newest counterpart the Single Cell Expression Atlas (www.ebi.ac.uk/gxa/sc) are EMBL-EBI’s knowledgebases for gene and protein expression and localisation in bulk and at single cell level. These resources aim to allow users to investigate their expression in normal tissue (baseline) or in response to perturbations such as disease or changes to genotype (differential) across multiple species. Users are invited to search for genes or metadata terms across species or biological conditions in a standardised consistent interface. Alongside these data, new features in Single Cell Expression Atlas allow users to query metadata through our new cell type wheel search. At the experiment level data can be explored through two types of dimensionality reduction plots, t-distributed Stochastic Neighbor Embedding (tSNE) and Uniform Manifold Approximation and Projection (UMAP), overlaid with either clustering or metadata information to assist users’ understanding. Data are also visualised as marker gene heatmaps identifying genes that help confer cluster identity. For some data, additional visualisations are available as interactive cell level anatomograms and cell type gene expression heatmaps.

## Graphical Abstract

**Graphical Abstract:**
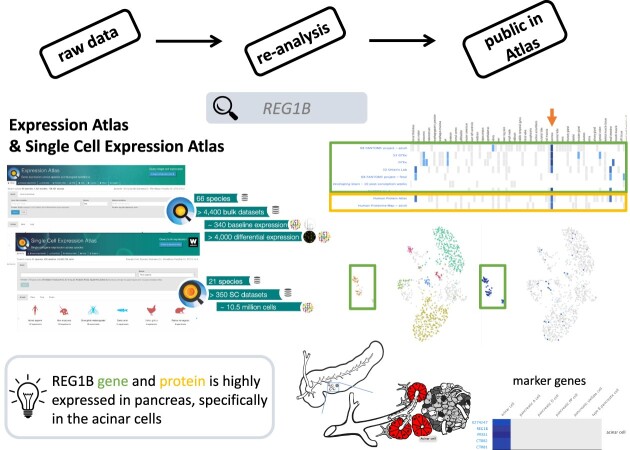

## Introduction

Expression Atlas (1) (https://www.ebi.ac.uk/gxa) and the Single Cell Expression Atlas (https://www.ebi.ac.uk/gxa/sc) are knowledgebases (added-value resource) for consistently analysed gene and protein expression data that are developed and maintained by the Gene Expression team at the European Molecular Biology Laboratory's European Bioinformatics Institute (EMBL-EBI). Data provided in the Expression Atlases are aimed at allowing users to investigate the localisation and abundance of gene and protein expression at the bulk and single cell level. Presenting these data in a consistent interface across resources allows users to investigate gene expression data across technologies (bulk sequencing, microarray, well-based and multiplexed single cell sequencing) to gain consensus and insight from publicly available and controlled access data in a single resource.

Datasets are sourced from public archives, such as BioStudies (2), PRIDE (3), NCBI’s Gene Expression Omnibus (GEO) (4), the European Nucleotide Archive (ENA) (5), dbGaP (6) and the European Genome-Phenome Archive (EGA) (7). With the continuous advancements in single-cell technologies and the increasing availability of data from a wider array of organisms, Single Cell Expression Atlas has increased its coverage of scRNA-seq datasets from various plant species and cell atlas projects, such as the Fly Cell Atlas (8).

In addition to transcriptomics data, Expression Atlas has integrated protein expression information in the same web interface alongside the gene expression data. Public mass spectrometry (MS)-based proteomics datasets coming mainly from the PRIDE database are selected, manually curated and reanalysed. For enabling data integration, protein expression results are reported in gene coordinates.

With the increase in datasets within Single Cell Expression Atlas and the addition of new species, the user interface has been improved to accommodate for metadata searches in addition to gene searches. Moreover, the results of metadata searches (including cell type search) can be viewed in a summarised way, highlighting the coverage of data that matches these keywords and cell types across different species. Top-scoring genes can be easily viewed across studies and species, providing a powerful way for easy interpretation by the scientific research community.

All data and analysis pipelines are designed to incorporate FAIR data principles (9) (Findable, Accessible, Interoperable, Reusable) to allow for their reuse and uptake by the scientific community. Data ingestion focuses on structuring metadata and mapping to ontology terms (controlled vocabularies) with input from species specific and subject matter experts (SMEs) where appropriate. This work aims to richly describe the entities represented in the resource and allow comparison of the same uniquely identified entity (such as cell type) across multiple datasets. Additionally, we apply these principles to our analysis workflows so that users can freely access and reuse the tools and processes developed by the team in their own work with a greater understanding of how data is derived. Lastly, as part of the EMBL-EBI’s access policy all data visualisations and processed data are also made available under a CC0 licence.

## Main updates

### Datasets and species update

From their inception, we aim to consistently improve the quality and quantity of datasets in both Expression Atlas and Single Cell Expression Atlas. The latest release of Expression Atlas in July 2023 focuses on increasing the quality of data presented to users. The resource contains 4424 datasets comprising 15 840 assays. The inclusion of data from a new species *Drosophila pseudoobscura* brings the total of represented organisms in the knowledgebase to 66. Of these, we also continue to increase the representation of proteomics data (93 datasets) and the inclusion of baseline expression data comprising 340 experiments across 47 different organisms.

Single Cell Expression Atlas has also focused on increasing the coverage of species and the inclusion of new data into the resource. As of its latest release in March 2023 the knowledgebase contains 355 datasets across 21 species. These data are derived from over 17 million cells, of which 10.5 million passed our quality checks and are displayed for users to explore, through dimensionality reduction plots (t-SNE and UMAP) as well as gene expression heatmaps and marker gene identification. We also include a new species in this release, *Xenopus tropicalis*, increasing the representation of single cell data from multiple species.

The top 10 species represented by experiments in Expression Atlas and Single Cell Expression Atlas are summarised in (Table 1).

**Table 1.** Top 10 species represented by experiment number in Single Cell Expression Atlas and Expression Atlas

Species	Single Cell Expression Atlas	Expression Atlas	Proteomics
*Homo sapiens*	146	1600	64
*Mus musculus*	122	1273	20
*Drosophila melanogaster*	29	150	
*Danio rerio*	14	26	
*Arabidopsis thaliana*	13	625	
*Gallus gallus*	4	40	
*Rattus norvegicus*	3	186	9
*Zea mays*	2	89	
*Oryza sativa Japonica Group*	2	112	
*Saccharomyces cerevisiae*	1	49	
**Species**	**Single Cell Expression Atlas**	**Expression Atlas**	
*Arabidopsis thaliana*	13	625	
*Zea mays*	2	89	
*Oryza sativa*	3	112	
*Glycine max*		22	
*Triticum aestivum*		19	
*Vitis vinifera*		33	
*Sorghum bicolor*		11	
*Solanum lycopersicum*	2	20	
*Hordeum vulgare*		16	
*Medicago truncatula*		10	

Additional table representing the scarcity of single cell sequencing data in plants compared to the top 10 plant species represented in Expression Atlas.

### Proteomics data

In the last two years, we have continued to increase the content of proteomics datasets in Expression Atlas, in collaboration with the PRIDE team at EMBL-EBI. Expression Atlas now includes protein expression results, which have been further refined, coming from 93 proteomics datasets. The datasets, all of them generated using label-free techniques, can be split in two main groups according to the type of proteomics data acquisition:

- Datasets generated using Data Dependent Acquisition (DDA) approaches. In this case, MaxQuant (10) was used as the analysis software, followed by an in-house post-processing pipeline. The integration of three groups of baseline tissue-based datasets was finalised, coming from human (32 organs represented) (11), mouse (13 organs) and rat (8 organs) (12) samples. To complement these, a study including 15 datasets coming from farm pig (*Sus scrofa*) (14 organs) has just been finalised and is now being integrated (not yet finalised at the moment of writing). In addition to baseline tissue data, 12 datasets coming from colorectal cancer samples have been reanalysed and integrated (datasets in Expression Atlas tagged as ‘ColCancer2023’), enabling the detection of biomarkers at the protein level. This is a continuation of our previous efforts in cell lines and tumour tissue (13).
- Datasets generated using Data Independent Acquisition (DIA) approaches. We performed a pilot project to study the feasibility of performing a systematic reanalysis of DIA datasets and included cell-line, human cancer-related and plasma samples (14). At the time, a spectral library based approach using the ‘Pan Human library’ was used; these datasets are tagged as ‘DIAPilot2021’. At present we are benchmarking a library-free methodology using DIA-NN (15), and applying it to additional datasets generated from human baseline tissues as a starting point. These datasets will get integrated into Expression Atlas in the near future, once the analyses are finalised.

Data integration between transcriptomics and proteomics datasets is enabled because protein expression data is reported in a gene-centric manner.

### Standardisation of analysis workflow

In the last two years we have shifted most of our analysis pipelines to modern workflow managers (Snakemake (16), Nextflow (17), Galaxy (18)), which could be portable and easily cloud deployed, with automatic dependency resolution. Migration to a more modern, community maintained, explicit workflow environment enables a faster turn-over in terms of variations to the analysis workflows, execution on multiple environments, granular tool updates and in general make the workflow more maintainable and continue our adherence to incorporating FAIR (9) principles into our pipelines. Because of the workflow modernisation achieved, the transition of our pipelines between different environments is straightforward, not only facilitating deployment in HPC and cloud environments, but also smoother release cycles and the continuation of the service in the future.

### Improvement of bioinformatics pipelines

In terms of bioinformatics tools update, we have adapted the Single Cell Expression Atlas droplet quantification workflow, which runs under Nextflow (17) for single-nucleus RNA sequencing experiments (snRNA-seq), which presented low alignment rates. We performed an internal benchmark and analysed the impact of different references and tools (alevin (19), alevin-fry (20), kallisto|bustools (21) and STARsolo (22)) on the mapping rates. In addition to increasing the alignment rate for snRNA-seq experiments, in corroboration with the original publication (20) we observed that alevin-fry was faster and required less memory. Therefore, we decided to switch from alevin to alevin-fry with unspliced (intron-containing) transcripts references for quantification of droplet based experiments.

### User interface

#### Data curation

At the heart of all data that gets ingested into Expression Atlas and Single Cell Expression Atlas is the incorporation of FAIR principles and data curation. From the onset, data are identified by the curation team for their rich biological and technical metadata and file integrity. These are then curated so that all metadata available for the experiment at the sample and file level are identified, and incorporated into the dataset. Where possible, all metadata are mapped to the corresponding ontology (controlled hierarchical vocabulary) term and species specific ontology where applicable (e.g. *Drosophila melanogaster* metadata are mapped to the ‘Drosophila gross anatomy ontology’ (FBbt) maintained by the FlyBase (23) team).

In addition, we strive to contact authors for cell type specific information which is inferred directly from the cells’ expression profile. These are represented to users in the original format as provided by the data owner, termed authors cell type. A second mapping where terms are mapped by the curation team to the closest relevant ontology term is also provided and represented to users as ‘authors cell type – ontology labels’. These are overlaid as metadata onto the dimensionality reduction plots on the results page for every experiment where available. These data visualisations are also made freely available to users for download as high quality images for reuse and integration into their work.

The benefit of this ontology mapping is clear as it allows users to consolidate and investigate all data where cell types are identified across diverse species, tissues and datasets to look for common or cell type specific genes which may infer functionality. This functionality is also integral to the metadata search wheel visualisation described in this paper (see below).

#### Metadata search and cell type wheel

The latest feature for Single Cell Expression Atlas is the addition of a ‘metadata search’ option that allows users to investigate data annotated with a biological entity, such as an organism part, cell type or disease. The addition of this feature to Single Cell Expression Atlas mirrors the existing functionality in Expression Atlas and allows users to understand data in greater detail and assist in gaining insight into the data. The metadata search allows users to search for a biological entity from the search bar on the Single Cell Expression Atlas landing page. From there, a powerful ontology-mediated search expansion is applied so that a user's search encompasses all synonyms, spellings and ‘child terms’ (associated more specific terms for a search e.g. a search for cancer would include child terms such as lung cancer, glioblastoma etc.).

Once a search is submitted to the web browser, the results are presented in a ‘cell type wheel’. This presents the search to the user as a series of ‘layers’ in a wheel. The innermost entity being the search term, and corresponding rings increasing in specificity, from species, to tissue to finally the cell type associated with that entity in the outermost ring. Selecting each ring refactors the results to expand the next associated outer ring and display to the user in more detail the entities associated with that ring. Users can manipulate the search display by clicking on the ‘rings’ along the top of the page to go back through the specific layers and their previous search history within the metadata results for their search.

**Figure 1.**
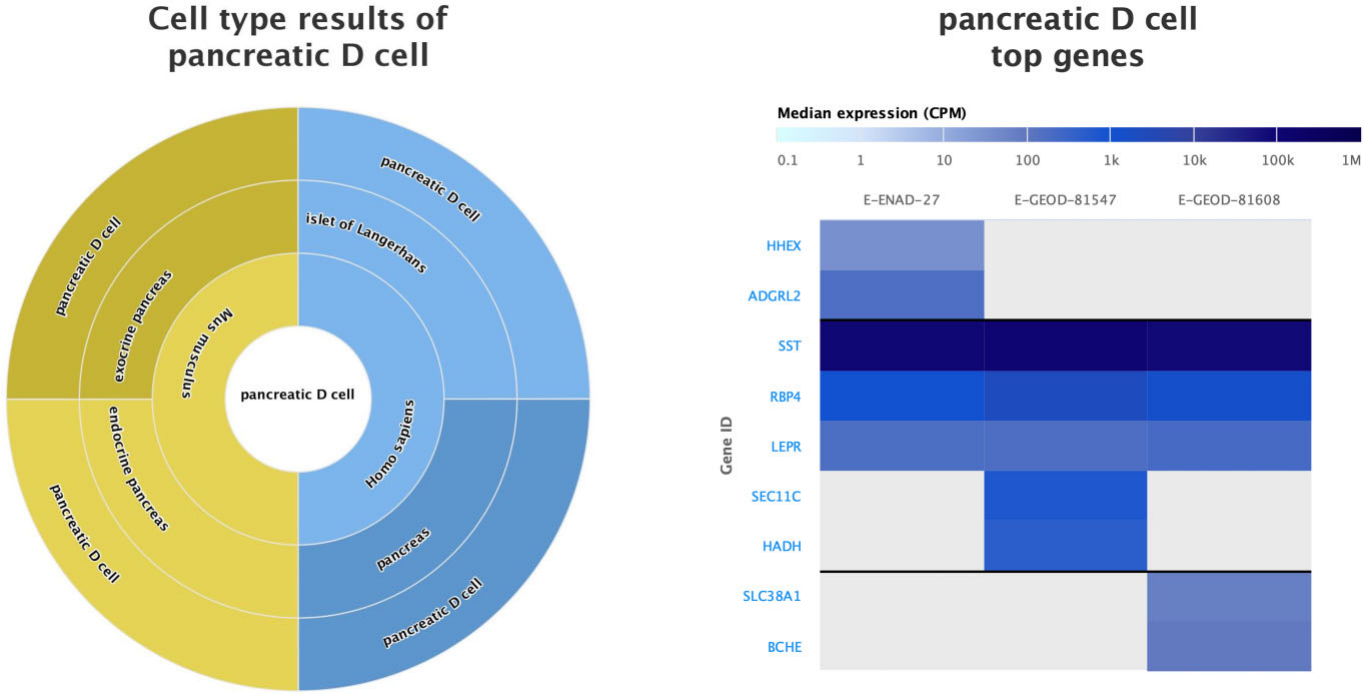
Cell type wheel visualisation of pancreatic D (delta) cell metadata search in Single Cell Expression Atlas alongside heatmap showing the top 5 genes across datasets for this cell type in *Homo sapiens* datasets.

Upon selecting an entity (cell type) in the outermost ring where possible, a cell type heatmap showing the top 5 genes associated with that cell type is displayed alongside the cell type wheel. This allows users to see all datasets where their particular entity (e.g. Paneth cell in pancreas in human datasets) have been investigated. Users can then understand broad consensus and differences in the top genes expressed for that entity (Figure 1). Again, these data visualisations can also be freely downloaded by users as high quality images for integration into their work.

#### Anatomograms expansion and inclusion of new species

**Figure 2.**
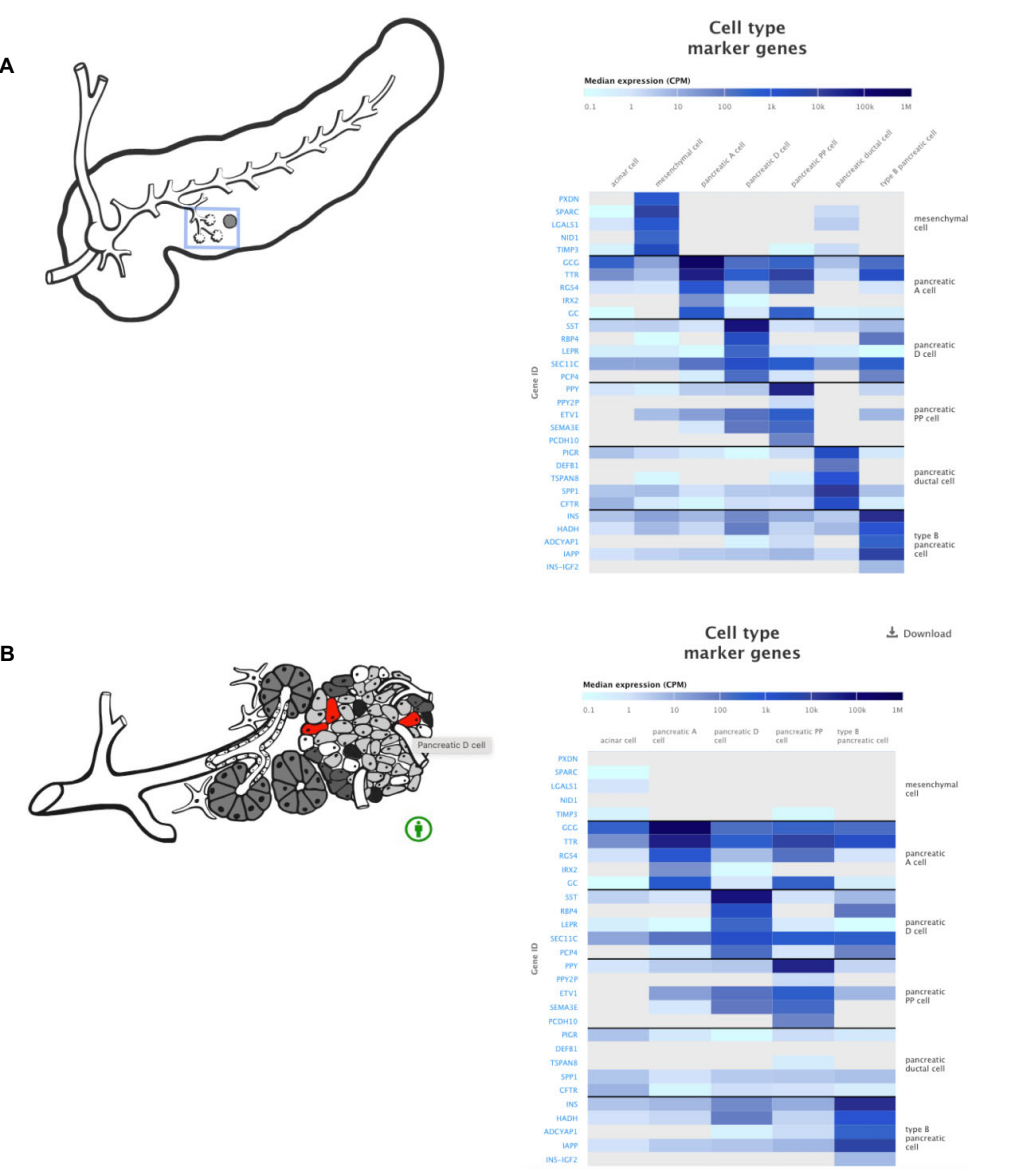
(**A**) The Single Cell Expression Atlas organ anatomogram for pancreas (e.g. https://www.ebi.ac.uk/gxa/sc/experiments/E-GEOD-83139/results/anatomogram), displaying marker genes for the different pancreatic cell types. Hovering over sections of the heatmap gives details about the gene's expression. As the user clicks on an active section of the pancreas anatomogram, the heatmap to the right changes to display only cell types that exist under that specific part of the organ. (**B**) As the user dives into more and more detailed views, it will end up at a cellular view.

As described in the previous Expression Atlas update paper (1), anatomograms are interactive single cell visualisations of cell types ‘in situ’ for healthy adult human tissue. These are developed through cross team collaborations between web developers, bioinformaticians, artists and data curators as well as SME’s in the scientific research community for that organ and species. The aim of these visualisations is to allow users to gain an ‘in situ’ understanding of organ structure and cell types alongside a ‘cell type heatmap’ that shows the top 5 marker genes specific to that cell type. An example of the use of anatomograms is shown in Figure 2, with a study on pancreatic cells.

In order to develop an anatomogram, in collaboration with research experts the curation and artists create an overview of the organ structure, including sub-organism and cell type configurations. These are represented as a structural series of images from top level macrostructures through a series of ‘zoom in’ images to microstructures and cell level architecture. Images are developed as shape layers on top of the base level image, representing the hierarchical nature of these tissues where cells are layered onto sub-tissue structures within the organ. This hierarchical nature is also recreated in the corresponding organ and cell type ontologies. Where required, additional cell type and sub-tissue structures and relationships missing from the relevant ontologies are identified, defined and added to the relevant ontology through community collaborations. In this way the tissue is represented both visually and hierarchically. All structures are then mapped to the corresponding image and ontology term prior to implementation by the web development team.

To link an image shape (e.g. cell type) to the corresponding data we again leverage the power of metadata curation. Curated datasets where possible are mapped to ontology terms, including as described earlier, inferred cell type information. These mappings correspond to the ontology shape mapping defined in the anatomogram. This linkage allows us to create a link between the shape and the corresponding expression profile for that entity and the cell type heatmap.

We continue to develop anatomograms and as part of these efforts, we have developed these visualisations for the organs related to the anatomy of the gut. Therefore in collaboration with the Human Gut Cell Atlas (24), we have developed a series of anatomograms representing the whole healthy adult human digestive tract, as well as a series representing the composite elements, including the colon, large and small intestines, mesenteric lymph nodes and anus.

## Discussion

### Community curation

Since their inception, both Expression Atlas and the Single Cell Expression Atlas have been committed to incorporating public datasets and where possible, controlled access, large scale and selected consortia data for use by the scientific community. This involves working with species and project communities to identify and incorporate data of value to their members. Additionally, we work with these community experts to ensure that the data incorporated aligns with their standards, ontologies and any additional requirements.

Through collaborating with these communities (Plant Cell Atlas, Fly Cell Atlas, Human Cell Atlas, European Diagnostic Transcriptomic Library (EDTL) and more) we are one of the few resources to contain multiple datasets across multiple species. This is particularly relevant for the plant community where single cell data is difficult to obtain, (Table 1).

Another element where community engagement is essential is the incorporation of inferred cell types. These are cell types conferred by investigation of their transcriptional profile as opposed to their biological characteristics, which have been the main source of cell classification. By reaching out to data owners we aim to include this information as often as possible for their dataset. Inferred cell type information are incorporated into the data visualisation both as metadata overlay on the dimensionality reduction plots and as mentioned previously for selected datasets in the anatomograms and cell type heatmap (see the relevant sections). Ontology mapping where possible is done in collaboration with a community expert and the corresponding ontology is updated where required to classify these new cell types.

The addition of inferred cell type identity to these data has been instrumental in future work, such as the cell type deconvolution of existing bulk data (see the deconvolution section below) as well as any future pipelines aiming to programmatically identify cells from existing expression profiles.

Future work in the team aims to make the process of community engagement as easy as possible with the aim of encouraging users to submit data directly to the Atlases as a potential endpoint. This would help resources continue to ingest data in line with the increase in publications which far outstrips the ability of manual curation teams to do so. With this in mind we aim to make the process of converting datasets from existing International Nucleotide Sequence Database Collaboration, INSDC, (The International Nucleotide Sequence Collaboration, https://www.insdc.org/) resources, ENA (25), Sequence Read Archive, SRA (26) and DNA Data Bank of Japan, DDBJ, (27) formats into MAGE-TAB (28) an automated process, requiring only the corresponding dataset accession. This is possible due to the shared data model across these resources and the alignment of this to the MAGE-TAB model. These MAGE-TAB would then be annotated by the community with ontology terms where possible and submitted for curation review. For single cell transcriptomic data for Single Cell Expression Atlas we would also encourage users to submit the corresponding inferred cell type data in a standardised format for inclusion to their dataset.

We have already started on this process, partly through the improvement of existing scripts which query the ENA API (Application Programming Interface) to convert both GEO and ENA data into MAGE-TAB format programmatically rather than manually, significantly reducing manual effort. We have also worked extensively with community partners and training events in data and knowledge management, MAGE-TAB structure and the inclusion of ontology terms to train and pass on our knowledge and tools to these communities. Our aim is to allow these communities to have these tools at their disposal and promote their reuse to their communities so that data comes directly from them rather than identified by the curation team through publication searches which are not extensive.

### Single cell expression atlas and community resources

#### FlyBase

As part of our commitment to working with communities, we also make data available to feed back into community repositories. For the FlyBase collaboration, data from Single Cell Expression Atlas are incorporated into this repository. The point of this is to fulfil three main aims: (i) to help FlyBase users to *discover* what data and datasets are available; (ii) provide information about relevant datasets and (iii) get a quick overview of expression data from these datasets. To assist with this, Single Cell Expression Atlas provides firstly, additional metadata about samples from the manual curation of datasets. Secondly, Single Cell Expression Atlas provides data matrices, containing gene expression per cell alongside the inferred cell type identity. With these data, FlyBase extracts i. the extent of expression ie. the proportion of cells of a given cell type in that dataset in which a gene is detected and ii. The average expression (normalised to CPM) in cells of that type which do express that gene.

**Figure 3.**
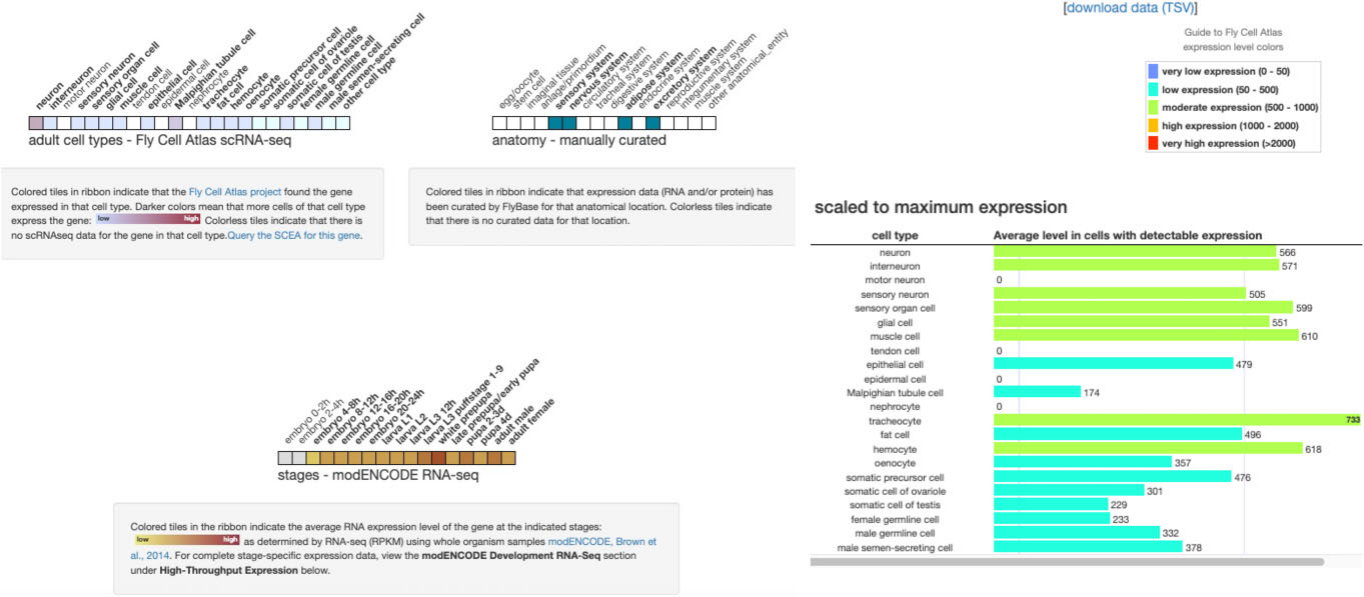
FlyBase results for *Dmel/w* showing the cell type ribbon, where tiles are coloured by the extent of expression of *Dmel/w* in a range of cell types from the dataset alongside a graphical display of high throughput expression data as a bar graph corresponding to both the proportion of cell types which express *Dmel/w* alongside the average expression of that gene in those cell types.

For a particular dataset record in FlyBase, users are provided with links to the corresponding datasets in Single Cell Expression Atlas. In the specific case of the Fly Cell Atlas dataset (8), users can also explore the data for a given gene using either the cell type ribbon, where tiles are coloured by the extent of expression of that gene in a range of cell types from the dataset or a graphical display of high throughput expression data as a bargraph corresponding to both the proportion of cell types which express the gene alongside the average expression of that gene in those cell types (Figure 3) (23).

An additional expansion to this project is the development of anatomograms for healthy adult tissue from *Drosophila melanogaster* as part of the community Fly Cell Atlas project and the FlyBase curation team. Initial development includes anatomograms for reproductive organs, the ovary and testis as well as a representation of the whole adult fly and composite organs.

#### Gramene

Another long standing collaboration between Expression Atlases and the plant community is our collaboration with the Gramene project (29). Expression data derived from plant data ingested into the Atlases is dynamically represented and updated via an embedded Atlas widget within Gramene's search browser with future plans to include Single Cell Expression data at the cellular level. We also work closely with the Gramene team to identify and ingest key datasets of interest to the plant community for both bulk and single cell expression.

#### Cell-type deconvolution

Deconvolution of RNA-seq experiments in Atlas have been implemented based on the recommendations of Vathrakokoili-Pournara *et al.* (30) using a set of organism part-specific references from the Single Cell Expression Atlas. Three selected deconvolution tools implemented in R (DWLS (31), FARDEEP (32), EpiDISH (33)) are run for bulk RNA-seq experiments from human, mouse and fruit fly. The estimated deconvolution results are reported if the mean Pearson correlation between the output cell proportion matrices is equal or higher than 0.6 to ensure robustness of the predictions. In the future, the results provided by this analysis will deliver Atlas users with additional information about estimated cellular heterogeneity of bulk samples. The user will then be linked back to the cell type wheel of the respective organism part and cell type in Single Cell Expression Atlas.

#### Proteomics

In the case of label-free quantitative proteomics datasets, the main focus in the proteomics field is shifting to DIA approaches, thanks to advances in instrumentation and computational analysis. One of the effects in DIA datasets is the reduction of missing values. DDA MS2-labelled approaches as TMT (Tandem Mass Tagging) remain also popular, although they are preferred for differential studies. However, methodology has been recently developed to represent these datasets as baseline data (34). In addition to bulk tissue data, single cell proteomics datasets are being generated at an increasing pace (35) although the instrumentation required makes this possible only for a small number of groups still. Although many of these datasets are still generated for method development purposes mainly, we anticipate a higher number of biologically relevant ones. We will attempt to use the ‘Single Cell Expression Atlas’ for providing access to these datasets.

## Data Availability

Expression Atlas and Single Cell Expression Atlas are available for users at https://www.ebi.ac.uk/gxa/ and at https://www.ebi.ac.uk/gxa/sc/, respectively. The Expression Atlas web application is open source and available in the GitHub repositories https://github.com/ebi-gene-expression-group/atlas-web-single-cell DOI: 10.5281/zenodo.10021406, https://github.com/ebi-gene-expression-group/atlas-web-bulk DOI: 10.5281/zenodo.10021638 and https://github.com/Papatheodorou-Group/CATD_snakemake DOI: 10.5281/zenodo.10021678 among others. The Nextflow pipeline to perform benchmark of different tools and references for snRNA-seq datasets is available at https://github.com/ebi-gene-expression-group/snRNA-mapping-rate DOI 10.5281/zenodo.10021661.
